# Fragmented population structure of *Plasmodium falciparum* in Papua New Guinea: Implications for malaria control

**DOI:** 10.1186/1475-2875-11-S1-P113

**Published:** 2012-10-15

**Authors:** Alvssa E Barry, Ivo Mueller, GL Abby Harrison, Celine Barnadas, Inoni Betuela, Manuel Hetzel, Livingstone Tavul, Dominic Kwiatkowski, Peter M Siba

**Affiliations:** 1 Infection and Immunity Division, Walter and Eliza Hall Institute, Melbourne, Australia; 2 Department of Medical Biology, University of Melbourne, Melbourne, Australia; 3 Centro de Investigación en Salud Internacional de Barcelona (CRESIB), Barcelona, Spain; 4 Papua New Guinea Institute of Medical Research, Goroka, PNG; 5 Sanger Institute, Hinxton, UK

## 

Malaria is being controlled in Papua New Guinea (PNG) where the epidemiology of the disease ranges from highly endemic in low-lying regions to epidemics in the highlands. Analyses of microsatellite haplotypes have revealed that populations of *Plasmodium falciparum* on the north coast of PNG are genetically isolated. If this fragmented population structure is found throughout PNG it will provide a unique opportunity for planning malaria control strategies and focusing efforts on regions where they are likely to have the greatest impact. We are working towards defining a high-resolution population genomic map of parasite networks and migration patterns throughout PNG using single nucleotide polymorphisms. Our approach, preliminary data and the practical implications of this research will be discussed in context with the national malaria control program.

